# Diethyl 2-[(*N*-benzyl-*N*-methyl­amino)(phen­yl)meth­yl]propane­dioate

**DOI:** 10.1107/S1600536810007506

**Published:** 2010-03-06

**Authors:** Ihssan Meskini, Maria Daoudi, Jean-Claude Daran, Hafid Zouihri, Taibi Ben Hadda

**Affiliations:** a Laboratoire de Chimie Organique, Faculté des Sciences Dhar el Mahraz, Université Sidi Mohammed Ben Abdellah, Fès, Morocco; b Laboratoire de Chimie de Coordination, 205 Route de Narbonne, 31077 Toulouse Cedex, France; c Centre National pour la Recherche Scientifique et Technique, Division UATRS, Rabat, Morocco; d Laboratoire de Chimie des Matériaux, Université Med. 1ier, Oujda, Morocco

## Abstract

In the title compound, C_22_H_27_NO_4_, the mean planes of the two benzene rings form a dihedral angle of 73.54 (13)°. One of the methyl groups is disordered over two sites, with site occupation factors of 0.47 (15) and 0.53 (15). The crystal packing is controlled by van der Waals forces and a possible C—H⋯O inter­action, forming a chain running parallel to the *a* axis.

## Related literature

For related compounds displaying biological activity, see: Dayam *et al.* (2007 ▶); Patil *et al.* (2007 ▶); Ramkumar *et al.* (2008 ▶); Sechi *et al.* (2009*a* ▶,*b* ▶); Zeng *et al.* (2008*a* ▶,*b* ▶). For the synthetic procedure, see: Pommier & Neamati (2006 ▶). For bond-length data, see: Allen *et al.* (1987 ▶).
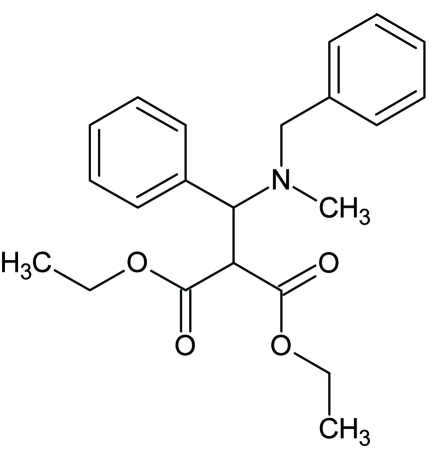


## Experimental

### 

#### Crystal data

C_22_H_27_NO_4_
*M* *_r_* = 369.45
Monoclinic, 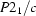

*a* = 9.3074 (2) Å
*b* = 5.9077 (1) Å
*c* = 37.0971 (7) Å
β = 92.999 (1)°
*V* = 2037.00 (7) Å^3^
*Z* = 4
Mo *K*α radiation
μ = 0.08 mm^−1^
*T* = 296 K
0.24 × 0.20 × 0.13 mm

#### Data collection

Bruker X8 APEXII CCD area-detector diffractometer
25575 measured reflections
4138 independent reflections
3411 reflections with *I* > 2σ(*I*)
*R*_int_ = 0.038

#### Refinement

*R*[*F* ^2^ > 2σ(*F* ^2^)] = 0.070
*wR*(*F* ^2^) = 0.159
*S* = 1.25
4138 reflections
257 parameters
1 restraint
H-atom parameters constrained
Δρ_max_ = 0.27 e Å^−3^
Δρ_min_ = −0.26 e Å^−3^

### 

Data collection: *APEX2* (Bruker, 2005 ▶); cell refinement: *SAINT* (Bruker, 2005 ▶); data reduction: *SAINT*; program(s) used to solve structure: *SHELXS97* (Sheldrick, 2008 ▶); program(s) used to refine structure: *SHELXL97* (Sheldrick, 2008 ▶); molecular graphics: *PLATON* (Spek, 2009 ▶); software used to prepare material for publication: *publCIF* (Westrip, 2010 ▶).

## Supplementary Material

Crystal structure: contains datablocks I, global. DOI: 10.1107/S1600536810007506/pv2262sup1.cif

Structure factors: contains datablocks I. DOI: 10.1107/S1600536810007506/pv2262Isup2.hkl

Additional supplementary materials:  crystallographic information; 3D view; checkCIF report

## Figures and Tables

**Table 1:** Hydrogen-bond geometry (Å, °)

*D*—H⋯*A*	*D*—H	H⋯*A*	*D*⋯*A*	*D*—H⋯*A*
C32—H32*A*⋯O3^i^	0.96	2.38	3.273 (3)	155

Symmetry code: (i) 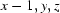.

**Fig. 1.**
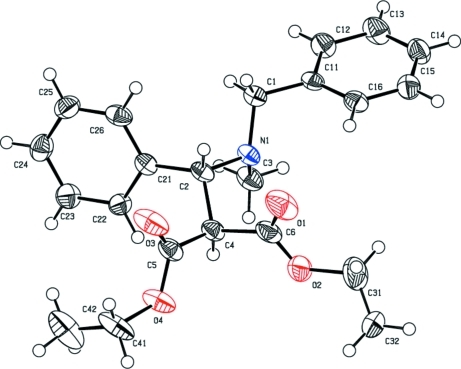
: Molecular structure of the title compound with the atom-labelling scheme. Displacement ellipsoids are drawn at the 50% probability level. H atoms are represented as small spheres of arbitrary radii.

**Fig. 2.**
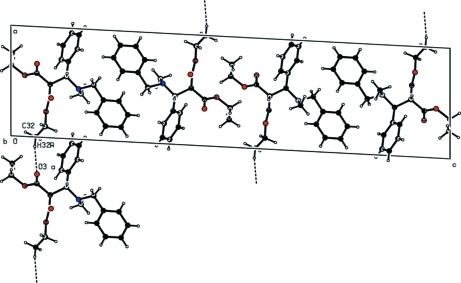
: Partial packing view showing the chain generated by C—H···O hydrogen bonds shown as dashed lines. [Symmetry code: (i) x-1,y,z].

## supplementary crystallographic information

### Comment

The rational design of new HIV-1 Integrase (H—I) inhibitors, one validated target for chemotherapeutic intervention (Dayam *et al.*, 2007), is fundamentally based on intermolecular coordination between H—I / chemical inhibitor / metals (Mg^+2^ and Mn^+2^, co-factors of the enzyme), leading in the formation of bimetallic complexes (Zeng *et al.*, 2008*a*; Sechi *et al.*, 2009*a*). Thereby, several bimetallic metal complexes, in many cases exploring the known-well polydentate ligands, appear in this scenario as the most promising concept to employ in either enzyme / drug interaction or electron transfer process, in the later case involving the biological oxygen transfer (Sechi *et al.*, 2009*b*; Ramkumar *et al.*, 2008). Another exciting example of application of such polydentate ligand involves the synergic water activation, that occurs via the so-called, remote metallic atoms. Such organometallic compounds are structurally deemed to promote or block the H—I activity (Zeng *et al.*, 2008*b*). The explanations given above clearly demonstrate that polydentate ligands are of special interest in the bioorganometallic chemistry (Patil *et al.*, 2007). Looking for the design of new bimetallic coordinating ligands to further explore in the building of intermolecular system involving H—I/ inhibitor/ metal complexation, we have targeted to study the crystallographic structure of a polydendate malonate N,*O*,*O*-ligand, the title compound (I).

In the molecule of (I) (Fig. 1), the benzene rings (C11—C16) and (C21—C26) are planar, with r.m.s deviation of 0.003 (3) Å and 0.009 (2) Å, respectively. The dihedral angle between the planes of these rings is 73.54 (13)°. The planes of the two carbonyl groups (O1,O2,C6) and (O3,O4,C5) are twisted by dihedral angles of 66.0 (3)° and 67.2 (3)°, respectively, with respect to (C4,C2,C21,N1) plane.

The Bond lengths and angles in the title compound have normal values (Allen *et al.*, 1987). The only significant intermolecular interaction in (I), as identified by PLATON (Spek, 2003) is a C32—H32A···O3 H-bond (Table 1).

### Experimental

The title compound, (I), was synthesized from 2-(benzylidene)-malonic acid diethyl esters in the presence of benzyl methylamine, in an aqueous medium at room temperature following the procedures reported earlier (Pommier & Neamati, 2006). To a solution of 2-(benzylidene)-malonic acid diethyl ester (1.24 g, 5 mmol) in water (20 ml) was added the benzyl methylamine (0.60 g, 6 mmol) and the stirring was continued at room temperature until the complete consumption of the starting material. After removing solvent, the crude products were dissolved in diethyl ether (2x40 ml) and washed with water until the pH became neutral. The organic solvent layer was dried with sodium sulphate and then evaporated. The residue was purified by recrystallization from a mixture  of diethylether/hexane (2:1) to give the pure compound (I) as colorless crystals in 78% yield.

### Refinement

All H atoms were fixed geometrically and treated as riding with C—H = 0.98 Å (methyne), 0.97 Å (methylene), 0.96Å (methyl) and 0.93Å (aromatic) with U_iso_(H) = 1.2U_eq_(C) or U_iso_(H) = 1.5U_eq_(C-methyl).

One of the methyl group is disordered over two positions with roughly identical occupation factors (0.47 and 0.53). These occupation factors were initially refined restraining their sum to be equal to 1 and applying an overall isotropic thermal parameter for the C atom. Moreover, C—C distances were restraint to have reasonable values. Once the occupation factors have been determined, they were fixed and not refined and the temperature factors were refined freely.

### Crystal data

**Table:**

C_22_H_27_NO_4_	*F*(000) = 792
*M**_r_* = 369.45	*D*_x_ = 1.205 Mg m^−^^3^
Monoclinic, *P*2_1_/*c*	Melting point = 380–382 K
Hall symbol: -P 2ybc	Mo *K*α radiation, λ = 0.71073 Å
*a* = 9.3074 (2) Å	Cell parameters from 3214 reflections
*b* = 5.9077 (1) Å	θ = 1.9–25.3°
*c* = 37.0971 (7) Å	µ = 0.08 mm^−^^1^
β = 92.999 (1)°	*T* = 296 K
*V* = 2037.00 (7) Å^3^	Block, colourless
*Z* = 4	0.24 × 0.20 × 0.13 mm

### Data collection

**Table:**

Bruker X8 APEXII CCD area-detector diffractometer	3411 reflections with *I* > 2σ(*I*)
Radiation source: fine-focus sealed tube	*R*_int_ = 0.038
graphite	θ_max_ = 26.4°, θ_min_ = 1.1°
φ and ω scans	*h* = −11→11
25575 measured reflections	*k* = −7→7
4138 independent reflections	*l* = −46→46

### Refinement

**Table:**

Refinement on *F*^2^	Primary atom site location: structure-invariant direct methods
Least-squares matrix: full	Secondary atom site location: difference Fourier map
*R*[*F*^2^ > 2σ(*F*^2^)] = 0.070	Hydrogen site location: inferred from neighbouring sites
*wR*(*F*^2^) = 0.159	H-atom parameters constrained
*S* = 1.25	*w* = 1/[σ^2^(*F*_o_^2^) + (0.0364*P*)^2^ + 1.9835*P*] where *P* = (*F*_o_^2^ + 2*F*_c_^2^)/3
4138 reflections	(Δ/σ)_max_ < 0.001
257 parameters	Δρ_max_ = 0.27 e Å^−^^3^
1 restraint	Δρ_min_ = −0.26 e Å^−^^3^

### Special details

**Table:**

Experimental. Yield = 78% (1.24 g). Rf = 0.45 (ether/hexane:1/1). Mp = 380-382 K.IR Spectroscopic (KBr, ν cm-1) analysis: 2860/2984 (C—H);1745 (CO); 1216/1301 (C—O); 1584/1601 (C=C), 1138, 1021.NMR analysis: ^1^H-NMR (250 MHz, CDCl_3_) δ (ppm): 7.2-7.4(m, 10H, aromat), 4.65 (d, H, Ph—C^3^H, ^3^J = 12.15 Hz), 4.01 (dq, 2H_AB_, OCH_2_CH_3_, ^2^J_AB_ = 14.34 Hz, ^3^J =7.06 Hz), 4.35 (dq, 2H_AB_, OCH_2_CH_3_, ^2^J_AB_ = 14.34 Hz,^3^J = 7.06 Hz), 4.32 (d, H, C^2^H(CO_2_Et)_2_ , ^3^J = 12.20 Hz), 3.5 (d, 1H, CH_2_Ph, ^3^J = 13.2 Hz), 3.25 (d, 1H, CH_2_Ph, ^3^J = 13.5 Hz), 2.05 (s, 3H, CH^3^), 1.33 (t, 3H, OCH_2_CH_3_, ^3^J = 7.2 Hz), 1.01 (t, 3H, OCH_2_CH_3_, ^3^J = 7.2 Hz). ^13^C-NMR (250 MHz, CDCl_3_) δ (ppm): 167.97 (C=O), 167.93 (C=O), 133.4 (C_quat_, C-(Ph), 132.6 (C_quat_, CPhCH_2_), 126.91/128.03 (C_tert_, 10CH aromt), 67.47 (C_tert_, C^3^HPh), 61.24 -61.3 (2 C, 2CH_2_CH_3_, ester), 55.39 (C_tert_, C^2^H-(CO_2_Et), 58.70 (C_ses_, C^ľ^H_2_N), 36.94 (C_ter_, CH_3_N), 14.15/13.73 ( 2 C, 2CH_3_, esters).. MS (IE) Calcd for [M]^+^ C_22_H_27_NO_4_: 369.45. [M+H]^+^. (m/z) = 370, [M—CH(CO_2_Et)_2_]^+^ (m/z) = 159 (100%).Elemental analysis for C_22_H_27_NO_4_ : Calc (Found): C 71.52 (71.48), H 7.37 (7.35), N 3.79 (3.81).The purity of the compound was checked by determining its melting point (380-382 K). Suitable single crystal of malonate derivative (I) was obtained by recrystallization from ethanol. A colorless crystal of (I) having approximate dimensions of 0.24 x 0.20 x 0.09 mm was mounted on a glass fibre. All measurements were made in the φ and ω scans technique on a CCD X8 Bruker diffractometer with graphite monochromatized MoKα radiation at room temperature (296 (2) K).The data collection nominally covered a sphere of reciprocal space, by a combination of five sets of exposures; each set had a different φ angle for the crystal and each exposure covered 0.5° in ω and 30 seconds in time. The crystal-to-detector distance was 37.5 mm.
Geometry. All esds (except the esd in the dihedral angle between two l.s. planes) are estimated using the full covariance matrix. The cell esds are taken into account individually in the estimation of esds in distances, angles and torsion angles; correlations between esds in cell parameters are only used when they are defined by crystal symmetry. An approximate (isotropic) treatment of cell esds is used for estimating esds involving l.s. planes.
Refinement. Refinement of *F*^2^ against ALL reflections. The weighted *R*-factor wR and goodness of fit *S* are based on *F*^2^, conventional *R*-factors *R* are based on *F*, with *F* set to zero for negative *F*^2^. The threshold expression of *F*^2^ > σ(*F*^2^) is used only for calculating *R*-factors(gt) etc. and is not relevant to the choice of reflections for refinement. *R*-factors based on *F*^2^ are statistically about twice as large as those based on *F*, and *R*- factors based on ALL data will be even larger.

### Fractional atomic coordinates and isotropic or equivalent isotropic displacement parameters (Å^2^)

**Table:**

	*x*	*y*	*z*	*U*_iso_*/*U*_eq_	Occ. (<1)
C1	0.4969 (3)	0.3979 (4)	0.18855 (6)	0.0302 (5)	
H1A	0.5442	0.5408	0.1841	0.036*	
H1B	0.5646	0.3016	0.2021	0.036*	
C2	0.5612 (3)	0.3238 (4)	0.12647 (6)	0.0290 (5)	
H2	0.5900	0.4832	0.1279	0.035*	
C3	0.4123 (3)	0.0546 (4)	0.15968 (7)	0.0391 (6)	
H3A	0.3777	−0.0094	0.1371	0.059*	
H3B	0.3374	0.0494	0.1765	0.059*	
H3C	0.4938	−0.0305	0.1691	0.059*	
C4	0.4827 (3)	0.2917 (4)	0.08947 (6)	0.0318 (6)	
H4	0.4493	0.1349	0.0870	0.038*	
C5	0.5794 (3)	0.3490 (5)	0.05896 (6)	0.0354 (6)	
C6	0.3536 (3)	0.4517 (5)	0.08739 (6)	0.0402 (7)	
C11	0.3667 (3)	0.4396 (4)	0.21050 (6)	0.0277 (5)	
C12	0.3634 (3)	0.3693 (5)	0.24598 (7)	0.0379 (6)	
H12	0.4411	0.2903	0.2565	0.045*	
C13	0.2448 (3)	0.4158 (5)	0.26603 (7)	0.0453 (7)	
H13	0.2434	0.3673	0.2899	0.054*	
C14	0.1298 (3)	0.5327 (5)	0.25081 (7)	0.0404 (7)	
H14	0.0509	0.5649	0.2643	0.048*	
C15	0.1318 (3)	0.6024 (5)	0.21538 (7)	0.0388 (6)	
H15	0.0536	0.6805	0.2049	0.047*	
C16	0.2495 (3)	0.5565 (4)	0.19540 (6)	0.0328 (6)	
H16	0.2500	0.6046	0.1715	0.039*	
C21	0.6990 (3)	0.1854 (4)	0.13072 (6)	0.0274 (5)	
C22	0.7136 (3)	−0.0263 (4)	0.11485 (6)	0.0306 (5)	
H22	0.6362	−0.0897	0.1015	0.037*	
C23	0.8421 (3)	−0.1431 (5)	0.11870 (7)	0.0380 (6)	
H23	0.8507	−0.2839	0.1078	0.046*	
C24	0.9581 (3)	−0.0524 (5)	0.13858 (7)	0.0396 (6)	
H24	1.0448	−0.1308	0.1408	0.048*	
C25	0.9443 (3)	0.1542 (5)	0.15501 (7)	0.0403 (7)	
H25	1.0213	0.2151	0.1688	0.048*	
C26	0.8162 (3)	0.2716 (5)	0.15109 (6)	0.0352 (6)	
H26	0.8082	0.4115	0.1623	0.042*	
C31	0.0998 (4)	0.4617 (8)	0.07836 (10)	0.0745 (12)	
H31A	0.0644	0.4759	0.1024	0.089*	0.53
H31B	0.1156	0.6124	0.0690	0.089*	0.53
H31C	0.0277	0.3731	0.0900	0.089*	0.47
H31D	0.1145	0.5999	0.0922	0.089*	0.47
C32	0.0021 (6)	0.3502 (13)	0.05651 (16)	0.0556 (17)	0.53
H32A	−0.0853	0.4364	0.0542	0.083*	0.53
H32B	−0.0173	0.2047	0.0667	0.083*	0.53
H32C	0.0398	0.3305	0.0331	0.083*	0.53
C32B	0.0454 (7)	0.5197 (15)	0.04332 (18)	0.059 (2)	0.47
H32D	0.0514	0.3908	0.0277	0.088*	0.47
H32E	0.1010	0.6418	0.0342	0.088*	0.47
H32F	−0.0533	0.5659	0.0443	0.088*	0.47
C41	0.6429 (4)	0.2245 (6)	0.00078 (8)	0.0617 (10)	
H41A	0.6915	0.3698	0.0009	0.074*	
H41B	0.5837	0.2120	−0.0214	0.074*	
C42	0.7512 (5)	0.0371 (8)	0.00302 (11)	0.0870 (13)	
H42A	0.8178	0.0623	0.0233	0.131*	
H42B	0.8025	0.0334	−0.0188	0.131*	
H42C	0.7029	−0.1047	0.0060	0.131*	
N1	0.4551 (2)	0.2901 (3)	0.15416 (5)	0.0284 (5)	
O1	0.3600 (3)	0.6519 (4)	0.09128 (6)	0.0592 (6)	
O2	0.2341 (2)	0.3345 (4)	0.07988 (5)	0.0529 (6)	
O3	0.6643 (3)	0.5002 (4)	0.05932 (5)	0.0576 (6)	
O4	0.5537 (2)	0.2080 (4)	0.03170 (5)	0.0509 (6)	

### Atomic displacement parameters (Å^2^)

**Table:**

	*U*^11^	*U*^22^	*U*^33^	*U*^12^	*U*^13^	*U*^23^
C1	0.0356 (13)	0.0276 (13)	0.0275 (12)	−0.0006 (11)	0.0029 (10)	−0.0026 (10)
C2	0.0402 (14)	0.0208 (12)	0.0263 (11)	0.0010 (11)	0.0052 (10)	−0.0036 (10)
C3	0.0504 (16)	0.0299 (14)	0.0380 (14)	−0.0036 (13)	0.0112 (12)	−0.0053 (12)
C4	0.0401 (14)	0.0275 (13)	0.0277 (12)	0.0078 (11)	0.0007 (10)	−0.0033 (10)
C5	0.0488 (16)	0.0314 (15)	0.0260 (12)	0.0143 (13)	0.0012 (11)	−0.0011 (11)
C6	0.0539 (18)	0.0454 (18)	0.0209 (12)	0.0153 (15)	−0.0006 (11)	−0.0032 (12)
C11	0.0350 (13)	0.0221 (12)	0.0260 (11)	−0.0042 (10)	0.0035 (9)	−0.0055 (10)
C12	0.0437 (15)	0.0389 (15)	0.0311 (13)	0.0070 (13)	0.0022 (11)	0.0039 (11)
C13	0.0549 (17)	0.0533 (19)	0.0288 (13)	0.0034 (15)	0.0119 (12)	0.0065 (13)
C14	0.0393 (15)	0.0461 (17)	0.0370 (14)	0.0000 (13)	0.0124 (11)	−0.0046 (13)
C15	0.0362 (14)	0.0424 (16)	0.0379 (14)	0.0048 (13)	0.0024 (11)	−0.0014 (12)
C16	0.0414 (14)	0.0329 (14)	0.0244 (11)	0.0009 (12)	0.0037 (10)	0.0009 (10)
C21	0.0357 (13)	0.0242 (12)	0.0229 (11)	0.0001 (11)	0.0060 (9)	0.0038 (9)
C22	0.0324 (13)	0.0269 (13)	0.0320 (12)	0.0010 (11)	−0.0028 (10)	−0.0006 (10)
C23	0.0425 (15)	0.0295 (14)	0.0417 (14)	0.0065 (12)	−0.0018 (12)	0.0001 (12)
C24	0.0337 (14)	0.0449 (17)	0.0400 (14)	0.0060 (13)	−0.0009 (11)	0.0090 (13)
C25	0.0338 (14)	0.0526 (18)	0.0343 (13)	−0.0098 (13)	−0.0006 (11)	−0.0007 (13)
C26	0.0465 (15)	0.0309 (14)	0.0287 (12)	−0.0108 (12)	0.0078 (11)	−0.0039 (11)
C31	0.059 (2)	0.104 (3)	0.060 (2)	0.048 (2)	−0.0056 (17)	−0.011 (2)
C32	0.030 (3)	0.085 (5)	0.051 (3)	0.008 (3)	−0.005 (3)	−0.014 (3)
C32B	0.035 (3)	0.081 (6)	0.061 (4)	0.010 (4)	0.004 (3)	0.024 (4)
C41	0.102 (3)	0.053 (2)	0.0321 (15)	0.002 (2)	0.0242 (16)	−0.0109 (14)
C42	0.115 (3)	0.082 (3)	0.068 (2)	0.019 (3)	0.041 (2)	−0.011 (2)
N1	0.0364 (11)	0.0226 (10)	0.0265 (10)	0.0002 (9)	0.0063 (8)	−0.0030 (8)
O1	0.0856 (17)	0.0391 (13)	0.0525 (13)	0.0256 (12)	0.0007 (11)	−0.0037 (10)
O2	0.0466 (12)	0.0626 (15)	0.0481 (12)	0.0262 (11)	−0.0087 (9)	−0.0141 (11)
O3	0.0867 (16)	0.0475 (13)	0.0407 (11)	−0.0182 (13)	0.0234 (11)	−0.0123 (10)
O4	0.0759 (15)	0.0480 (13)	0.0296 (9)	−0.0057 (11)	0.0112 (9)	−0.0109 (9)

### Geometric parameters (Å, °)

**Table:**

C1—N1	1.460 (3)	C21—C22	1.392 (3)
C1—C11	1.515 (3)	C22—C23	1.382 (3)
C1—H1A	0.9700	C22—H22	0.9300
C1—H1B	0.9700	C23—C24	1.383 (4)
C2—N1	1.475 (3)	C23—H23	0.9300
C2—C21	1.522 (3)	C24—C25	1.373 (4)
C2—C4	1.533 (3)	C24—H24	0.9300
C2—H2	0.9800	C25—C26	1.380 (4)
C3—N1	1.464 (3)	C25—H25	0.9300
C3—H3A	0.9600	C26—H26	0.9300
C3—H3B	0.9600	C31—C32	1.357 (6)
C3—H3C	0.9600	C31—C32B	1.412 (7)
C4—C5	1.521 (4)	C31—O2	1.457 (4)
C4—C6	1.528 (4)	C31—H31A	0.9700
C4—H4	0.9800	C31—H31B	0.9700
C5—O3	1.192 (3)	C31—H31C	0.9700
C5—O4	1.323 (3)	C31—H31D	0.9700
C6—O1	1.192 (3)	C32—H31C	1.2600
C6—O2	1.327 (4)	C32—H32A	0.9600
C11—C12	1.382 (3)	C32—H32B	0.9600
C11—C16	1.385 (3)	C32—H32C	0.9600
C12—C13	1.390 (4)	C32B—H32D	0.9600
C12—H12	0.9300	C32B—H32E	0.9600
C13—C14	1.370 (4)	C32B—H32F	0.9600
C13—H13	0.9300	C41—O4	1.454 (3)
C14—C15	1.378 (4)	C41—C42	1.497 (5)
C14—H14	0.9300	C41—H41A	0.9700
C15—C16	1.381 (4)	C41—H41B	0.9700
C15—H15	0.9300	C42—H42A	0.9600
C16—H16	0.9300	C42—H42B	0.9600
C21—C26	1.392 (3)	C42—H42C	0.9600
			
N1—C1—C11	110.89 (19)	C23—C24—H24	120.2
N1—C1—H1A	109.5	C24—C25—C26	120.0 (2)
C11—C1—H1A	109.5	C24—C25—H25	120.0
N1—C1—H1B	109.5	C26—C25—H25	120.0
C11—C1—H1B	109.5	C25—C26—C21	121.5 (2)
H1A—C1—H1B	108.0	C25—C26—H26	119.3
N1—C2—C21	116.50 (19)	C21—C26—H26	119.3
N1—C2—C4	107.57 (19)	C32—C31—C32B	51.0 (4)
C21—C2—C4	112.69 (19)	C32—C31—O2	108.7 (4)
N1—C2—H2	106.5	C32B—C31—O2	115.3 (4)
C21—C2—H2	106.5	C32—C31—H31A	110.0
C4—C2—H2	106.5	C32B—C31—H31A	134.6
N1—C3—H3A	109.5	O2—C31—H31A	110.0
N1—C3—H3B	109.5	C32—C31—H31B	110.0
H3A—C3—H3B	109.5	C32B—C31—H31B	60.1
N1—C3—H3C	109.5	O2—C31—H31B	110.0
H3A—C3—H3C	109.5	H31A—C31—H31B	108.3
H3B—C3—H3C	109.5	C32—C31—H31C	63.0
C5—C4—C6	108.6 (2)	C32B—C31—H31C	108.5
C5—C4—C2	111.5 (2)	O2—C31—H31C	108.5
C6—C4—C2	107.79 (19)	H31A—C31—H31C	50.3
C5—C4—H4	109.6	H31B—C31—H31C	140.9
C6—C4—H4	109.6	C32—C31—H31D	142.7
C2—C4—H4	109.6	C32B—C31—H31D	108.4
O3—C5—O4	125.0 (2)	O2—C31—H31D	108.5
O3—C5—C4	125.2 (2)	H31A—C31—H31D	59.1
O4—C5—C4	109.8 (2)	H31B—C31—H31D	52.9
O1—C6—O2	125.4 (3)	H31C—C31—H31D	107.5
O1—C6—C4	125.0 (3)	C31—C32—H31C	43.3
O2—C6—C4	109.6 (2)	C31—C32—H32A	109.5
C12—C11—C16	118.6 (2)	H31C—C32—H32A	98.3
C12—C11—C1	121.4 (2)	C31—C32—H32B	109.5
C16—C11—C1	119.9 (2)	H31C—C32—H32B	74.8
C11—C12—C13	120.6 (2)	C31—C32—H32C	109.5
C11—C12—H12	119.7	H31C—C32—H32C	147.7
C13—C12—H12	119.7	C31—C32B—H32D	109.5
C14—C13—C12	120.2 (2)	C31—C32B—H32E	109.5
C14—C13—H13	119.9	H32D—C32B—H32E	109.5
C12—C13—H13	119.9	C31—C32B—H32F	109.5
C13—C14—C15	119.6 (2)	H32D—C32B—H32F	109.5
C13—C14—H14	120.2	H32E—C32B—H32F	109.5
C15—C14—H14	120.2	O4—C41—C42	108.5 (3)
C14—C15—C16	120.3 (3)	O4—C41—H41A	110.0
C14—C15—H15	119.9	C42—C41—H41A	110.0
C16—C15—H15	119.9	O4—C41—H41B	110.0
C15—C16—C11	120.7 (2)	C42—C41—H41B	110.0
C15—C16—H16	119.6	H41A—C41—H41B	108.4
C11—C16—H16	119.6	C41—C42—H42A	109.5
C26—C21—C22	117.9 (2)	C41—C42—H42B	109.5
C26—C21—C2	119.5 (2)	H42A—C42—H42B	109.5
C22—C21—C2	122.6 (2)	C41—C42—H42C	109.5
C23—C22—C21	120.5 (2)	H42A—C42—H42C	109.5
C23—C22—H22	119.7	H42B—C42—H42C	109.5
C21—C22—H22	119.7	C1—N1—C3	110.74 (19)
C22—C23—C24	120.6 (3)	C1—N1—C2	113.09 (19)
C22—C23—H23	119.7	C3—N1—C2	114.98 (19)
C24—C23—H23	119.7	C6—O2—C31	116.5 (3)
C25—C24—C23	119.5 (3)	C5—O4—C41	118.2 (2)
C25—C24—H24	120.2		

### Hydrogen-bond geometry (Å, °)

**Table:**

*D*—H···*A*	*D*—H	H···*A*	*D*···*A*	*D*—H···*A*
C32—H32A···O3^i^	0.96	2.38	3.273 (3)	155

Symmetry codes: (i) *x*−1, *y*, *z*.
